# Techno-economic analysis of a power-to-hydrogen system in heavy industries with and without national incentives

**DOI:** 10.1371/journal.pone.0340602

**Published:** 2026-05-18

**Authors:** Ismail Marouani, Saleh Albadran, Mansoor Alturki, Yasser Almalaq, Badr M. Alshammari, Tawfik Guesmi

**Affiliations:** 1 Department of Electronics Engineering, Applied College, University of Ha’il, Ha’il, Saudi Arabia; 2 Department of Electrical Engineering, College of Engineering, University of Ha’il, Ha’il, Saudi Arabia; Indian Institute of Technology Kanpur, INDIA

## Abstract

This study analyzes how national incentive programs can play a transformative role in facilitating green hydrogen adoption across emission-intensive industrial sectors. Despite the recognized potential of green hydrogen for industry decarbonization, its widespread uptake remains constrained by elevated costs, limited supporting infrastructure, and technological limitations. By evaluating system optimization strategies, including PV-Wind, PEM electrolyser, energy storage and hydrogen tank sizing, this research demonstrates that targeted incentives applied to the redevelopment of legacy industrial zones can substantially reduce the Levelized Cost of Hydrogen (LCOH) from 7.8 USD/kg in baseline scenarios to 4.5 USD/kg with incentives considered, while simultaneously achieving notable reductions in greenhouse gas (GHG) emissions from approximately 3.2 kgCO₂eq/kgH₂ to near 1.4 kgCO₂eq/kgH₂. The novelty of this work is fourfold. It presents the first techno-economic optimization of Power-to-Hydrogen (PtH) systems that explicitly quantifies the interaction between national incentive schemes (0–70% CAPEX subsidies) and optimal sizing of PV, wind, electrolyzer, and hydrogen storage for heavy industrial applications. It demonstrates a linear relationship between total capital investment and LCOH (R² > 0.96), enabling rapid cost estimation without full simulations. It identifies a critical threshold for battery storage cost reduction (≥50%) before batteries become economically viable in PtH systems without incentives. It also provides a comparative analysis of incentive effects versus projected equipment cost reductions (2030–2050), showing that incentives alone can achieve 43–54% LCOH reductions. In addition, this formulated control strategy aims to accomplish three main objectives such as satisfying hourly hydrogen demand, maximizing renewable electricity utilization, and minimizing grid electricity withdrawal. The economic effect of these incentives closely rivals anticipated declines in equipment expenses projected for the coming decade. Furthermore, the observed linear relationship between capital investment and LCOH enables precise cost modelling and streamlines decision-making for site-specific implementations, minimizing the need for additional simulations.

## Introduction

The transition to a sustainable energy future is increasingly recognized as vital for mitigating climate change and reducing greenhouse gas emissions. Among various renewable energy sources, green hydrogen has emerged as a promising solution, particularly in heavy industries where traditional fossil fuels have long been the dominant energy source. The paper [1] explores the critical role of national incentives in enhancing the production of green hydrogen within heavy industrial sectors. Heavy industries, including steel, cement, and chemical production, are significant contributors to global carbon emissions, making their decarbonization crucial for achieving climate targets. However, the adoption of green hydrogen technologies faces substantial barriers, including high production costs, limited infrastructure, and technological challenges. National incentives such as subsidies, tax breaks, and supportive regulatory frameworks can play a transformative role. For instance, recent studies report that capital subsidies of 30–50% can reduce the LCOH significantly [2], while renewable electricity tariff exemptions lower operational expenses given that electricity costs can account for up to 70% of LCOH [3]. A general case study presented in [4] illustrates how effective national policies and incentives can drive strategic improvements in green hydrogen production, ultimately leading to enhanced sustainability and competitiveness in heavy industries. By analyzing various national approaches, the study aims to provide insights into best practices and policy recommendations that can accelerate the transition to a low-carbon economy. Recent literature has extensively explored various pathways for renewable and waste-to-energy hydrogen production. A global geospatial model integrating machine learning was developed to predict biogas and hydrogen potential from 9096 landfill sites, reporting that China can achieve a Levelized Cost of Hydrogen (LCOH) below 2.0 USD/kg from landfill gas [5]. Another study investigated hydrogen production from olive cake via combined air-steam gasification, using XGBoost machine learning and multi-objective optimization to achieve a hydrogen production rate of 329 kg/hr with an LCOH of 1.23 USD/kg and a payback period of 1.26 years [6]. An integrated assessment of green hydrogen production in California examined life cycle greenhouse gas emissions, techno-economic feasibility, and resource variability, finding that wind-based systems achieve lower carbon intensity (0.35–1.9 kg CO₂eq/kg H₂) compared to PV-based systems (1.58–2.95 kg CO₂eq/kg H₂) [7]. The integration of hybrid PV/wind-based electric vehicle charging stations with green hydrogen production in Kentucky was assessed, demonstrating that grid-connected hybrid systems achieve hydrogen production costs of approximately 6 USD/kg [8]. A polygeneration approach in wastewater treatment plants was evaluated for enhanced energy efficiency and green hydrogen/ammonia production, demonstrating the potential for industrial symbiosis in hard-to-abate sectors [9]. A geospatial techno-economic feasibility and life cycle greenhouse gas analysis for renewable hydrogen production across the Middle East and North Africa (MENA) region identified wind-powered hydrogen as generally cleaner and cheaper than PV-based production, with costs ranging from 2.0 to 7.0 USD/kg H₂ [10]. A global assessment of solar and wind-based renewable hydrogen production revealed that hybrid PV/wind systems maximize renewable energy utilization, achieving LCOH below 5 USD/kg H₂ in regions such as MENA, Australia, and the central United States [11]. Sustainable e-fuels production, liquefaction, and transport techno-economic feasibility using renewable energy resources in Egypt was examined, showing that Aswan and Hurghada achieve LCOH values of 6.29 and 5.00 USD/kg H₂, respectively [12]. Finally, life cycle and techno-economic insights into CO₂ capture and utilization for enabling e-fuels in the MENA region highlighted synergies between green hydrogen production and carbon management strategies [13]. The reference [14] investigates the transition from fossil fuel-based hydrogen production to renewable water electrolysis as a pathway toward efficient, low-carbon, and cost-effective hydrogen generation. The global demand for hydrogen is rising, driven by its potential as a clean energy carrier for various applications, including transportation, industry, and energy storage. However, traditional methods of hydrogen production, mainly reliant on fossil fuels, contribute significantly to greenhouse gas emissions. Renewable water electrolysis offers a sustainable alternative, using electricity from renewable sources to split water into hydrogen and oxygen. The paper provides an overview of the current state of electrolysis technologies, highlighting advancements in efficiency and scalability. It also examines the economic and environmental benefits of adopting renewable hydrogen production methods, stressing the importance of policy support and investment in infrastructure to facilitate this transition. The findings underscore the necessity for a coordinated approach that includes technological innovation, economic incentives, and regulatory frameworks to enable a shift toward sustainable hydrogen production. By addressing existing challenges and leveraging opportunities in renewable energy integration, the paper [14] outlines a comprehensive perspective and outlook for the future of hydrogen production, contributing to global decarbonization efforts and the establishment of a resilient hydrogen economy. The work presented in [15] investigates the potential of green hydrogen as a viable alternative to natural gas in power generation. As the world faces the urgent need to reduce greenhouse gas emissions and transition to sustainable energy systems, green hydrogen produced from renewable energy sources through water electrolysis emerges as a promising solution. This research evaluates the technical, economic, and environmental benefits of integrating green hydrogen into existing power generation frameworks, particularly in regions that heavily rely on natural gas. The analysis includes a comparison of lifecycle emissions associated with green hydrogen and natural gas, demonstrating the significant reduction in carbon footprints achievable through the adoption of hydrogen. Additionally, the paper explores the challenges of scaling up green hydrogen production, including infrastructure requirements and cost considerations, while highlighting technological advancements that can enhance its viability. By presenting case studies and current developments in the hydrogen sector, the paper emphasizes the necessity for supportive policies and investments to facilitate the transition from natural gas to green hydrogen. Ultimately, the findings advocate for the strategic incorporation of green hydrogen in power generation as a critical step toward achieving energy security, sustainability, and climate goals. The paper published by the International Energy Agency (IEA) [16], offers a comprehensive overview of clean energy transitions within the industrial sector. It highlights advancements in energy efficiency, renewable energy adoption, and innovative technologies designed to reduce carbon emissions. The report analyzes key sectors such as manufacturing, construction, and mining, providing insights into the unique challenges and opportunities they encounter. It identifies barriers to clean energy adoption, including high upfront costs and regulatory hurdles, and emphasizes the necessity for targeted policies and incentives. Furthermore, the IEA presents actionable recommendations for governments and industry stakeholders to accelerate this transition, along with forecasts for clean energy adoption. The report underlines that substantial efforts are still required to achieve the necessary reductions in greenhouse gas emissions. The reference [17] explores the potential of green hydrogen as a viable solution for meeting industrial heating needs. It examines the role of green hydrogen in decarbonizing high-temperature processes across various industries, emphasizing its advantages over traditional fossil fuels. The report analyzes the economic feasibility, technological advancements, and scalability of green hydrogen production, along with the infrastructure requirements for its integration into existing systems. Additionally, it discusses the regulatory landscape and market dynamics that influence the adoption of green hydrogen. Overall, the paper highlights the significant opportunities and challenges associated with leveraging green hydrogen to achieve sustainability goals in industrial heating. The study presented in [18] provides a comprehensive examination of the multifaceted challenges facing the green hydrogen sector in the United States. It discusses economic barriers such as high production costs and the need for investment in infrastructure, alongside social challenges including public perception and acceptance of green hydrogen technologies. The authors also explore regulatory hurdles, highlighting the complexities of existing policies and the lack of cohesive frameworks to support the growth of green hydrogen initiatives. Through this review, the paper emphasizes the importance of addressing these challenges to facilitate the successful integration of green hydrogen into the energy landscape, ultimately contributing to decarbonization efforts in various sectors. The reference [19] critically evaluates the capacity targets set for electrolysers within the framework of Italy’s Hydrogen Strategy. It examines the technical challenges associated with scaling up electrolyser production, including issues of efficiency and reliability. The authors discuss the economic implications, such as installation and operational costs, and the potential for achieving economies of scale. Additionally, the paper addresses environmental concerns, particularly regarding the lifecycle emissions of hydrogen production technologies. Through this analysis, the authors emphasize the need for a balanced approach that considers technical feasibility, economic viability, and environmental sustainability to effectively meet Italy’s hydrogen goals. The work [20] offers an in-depth analysis of the technological innovations and policy measures necessary to accelerate the adoption of green hydrogen. It examines advancements in electrolysis, hydrogen storage, and fuel cell technologies, while also assessing the impact of various policy frameworks on the growth of the green hydrogen sector. The authors argue that coordinated efforts in technology development and policy support are crucial for overcoming existing barriers and achieving large-scale deployment of green hydrogen solutions.

The study [21] provides a comparative evaluation of the green hydrogen industry’s development potential across China, the United States, and the European Union. The authors analyze key factors such as market dynamics, technological capabilities, and policy environments that influence the green hydrogen landscape in each region. The findings indicate that while all regions possess significant potential, their development paths differ: China prioritizes large-scale manufacturing to reduce electrolyzer costs, the US relies on production tax credits under the Inflation Reduction Act, and the EU emphasizes cross-border auctions and Hydrogen Valleys. These distinct approaches affect the pace and structure of green hydrogen deployment. In [22], the authors analyze existing rollout policies and propose a framework for optimizing financial support mechanisms. By examining case studies and policy outcomes, the paper highlights the critical role of targeted subsidies in fostering innovation and investment in the green hydrogen sector. The document [23] outlines Australia’s national strategy for hydrogen development, emphasizing the country’s commitment to becoming a leader in the global hydrogen market. It discusses the strategic priorities for hydrogen production, export, and domestic utilization, as well as the importance of collaboration between government, industry, and research sectors to drive innovation and achieve sustainability goals. A directive establishes a framework for promoting the use of renewable energy sources across the EU, focusing on enhancing sustainability and reducing greenhouse gas emissions [24]. It outlines targets for renewable energy integration, including specific provisions for bioenergy and hydrogen, thereby supporting the transition to a low-carbon economy. A regulation provides specific guidelines and requirements for implementing various EU policies related to renewable energy [25]. It includes provisions for monitoring and reporting on renewable energy usage, with particular emphasis on hydrogen as a key component of the EU’s energy transition strategy. The regulation aims to facilitate compliance and enhance the effectiveness of renewable energy promotion across member states. An announcement details the European Hydrogen Bank’s auction, which has allocated €720 million to support the development of green hydrogen projects across Europe [26]. The funding aims to stimulate investment in hydrogen infrastructure and production, enhancing the EU’s commitment to achieving its climate goals and transitioning to a sustainable energy system. A second renewable hydrogen auction, aimed at incentivizing the production of renewable hydrogen [27]. It provides details on eligibility, bidding processes, and the strategic objectives of the auction, emphasizing the EU’s efforts to bolster the hydrogen market as part of its broader renewable energy strategy. An initiative from the Italian Ministry of Environment and Energy Security focuses on producing hydrogen in disused industrial areas, known as Hydrogen Valleys [28]. It aims to revitalize these regions while promoting sustainable hydrogen production. The program outlines funding opportunities and specific projects designed to leverage existing industrial infrastructure for green hydrogen generation. The reference [29] discusses the PNRR M2C2 investment program in Piedmont, Italy, which targets hydrogen production in abandoned industrial sites, supported by EU Next Generation EU funds. It highlights the program’s goals to foster economic recovery and sustainability through innovative hydrogen projects, providing details on application processes and funding availability for interested stakeholders.

To bridge the existing research gaps, this study advances the techno-economic understanding and operational optimization of integrated Power-to-Hydrogen (PtH) systems through the following key contributions:

- A techno-economic optimization of PtH systems that explicitly quantifies the interaction between national incentive schemes (0–70% CAPEX subsidies) and optimal component sizing (PV, wind, electrolyzer, and hydrogen storage) for heavy industrial applications a contribution that has not been previously reported in the literature.
- A demonstrable linear relationship between total capital investment and LCOH (R² > 0.96 for both incentivized and non-incentivized scenarios), which enables rapid cost estimation without full simulations and streamlines site-specific decision-making.
- The identification of a critical cost-reduction threshold for battery storage (≥50%) before batteries become economically viable in PtH systems without incentives, along with an analysis of the interplay between battery size, electrolyzer capacity, and hydrogen storage.
- A comparative assessment of incentive effects versus projected equipment cost reductions (2030–2050), revealing that incentives alone can achieve 43–54% LCOH reductions, with combined effects potentially lowering LCOH to 2.9 USD/kg.

Additionally, this study performs a multi-scenario assessment of optimal PtH configuration under diverse renewable resource conditions, introduces a systematic modeling framework that quantifies the impact of fluctuating demand patterns, and evaluates different energy storage strategies within energy-intensive industrial environments.

## Methodology

### Optimization problem formulation

The objective is to minimize the LCOH subject to meeting hourly hydrogen demand. Decision variables are the rated capacities of PV (kWp), wind (kW), PEM electrolyzer (kW), battery (kWh), and hydrogen storage (kgH₂).

**Objective function:**


$$\begin{array}{c} min\;LCOH=\frac{\sum_{j}({CRF}_{j}\cdot {NPC}_{j}+{O\&M}_{j})+C_{grid}}{\sum_{i}m_{H\text{2}}(t)}\; \end{array}$$ (1)

**Constraints:**

Hourly hydrogen demand satisfaction

Battery SOC limits (0.1–0.9)

Hydrogen storage pressure limits (2–30 bar)

Electrolyzer power limits ($\overset{min}{\underset{El}{P}}$ to $\overset{max}{\underset{El}{P}}$)

Renewable curtailment allowed only after storage saturation

The optimization uses Particle Swarm Optimization (PSO) with 50 particles and 200 iterations.

### Hybrid system model description

**Fig 1:**
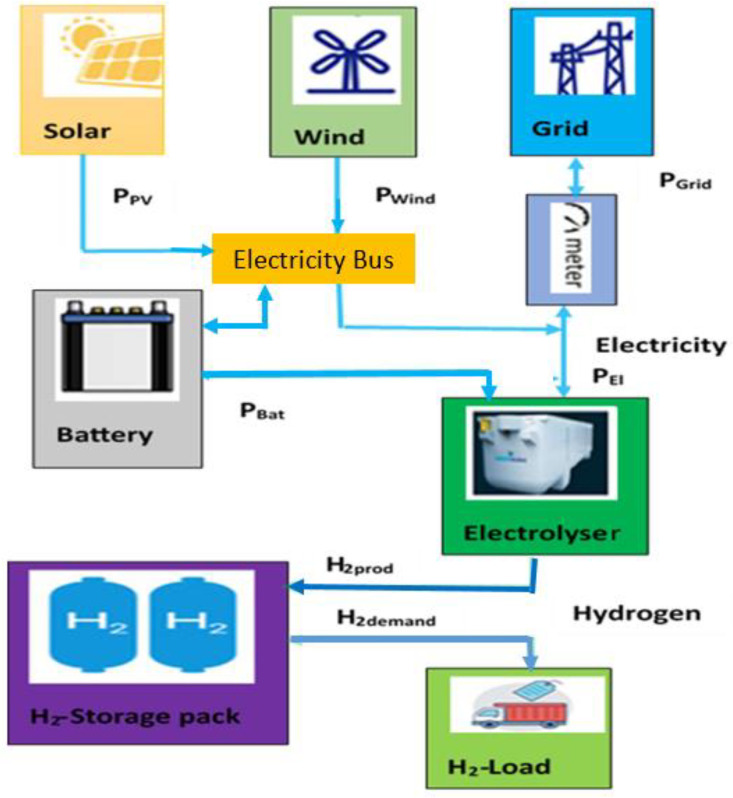
System configuration model.

A schematic overview of the investigated Power-to-Hydrogen setup is presented in Fig 1. In this system, renewable energy generated by both photovoltaic modules and wind turbines is allocated as input electricity for the electrolyzer, where water is converted to hydrogen. Energy storage is achieved using a lithium-ion battery, charged exclusively from renewable sources, to enable load shifting and enhance operational flexibility. Downstream from the electrolyzer, a pressurized hydrogen storage unit serves to retain produced hydrogen, increasing system adaptability. During periods of limited renewable output, the connection to the electrical grid allows supply to be supplemented, while excess renewable electricity when hydrogen storage and electrolyzer capacity are saturated can be diverted to the grid for efficient energy management. The H₂ Load represents the hourly hydrogen demand of the industrial consumer. The arrow from hydrogen storage to H₂ Load indicates that hydrogen is first stored and then supplied to meet demand, ensuring continuity during periods of low renewable generation.

A Python-based energy system model has been developed to represent the configuration shown in Fig 1. System component capacities were determined by applying Particle Swarm Optimization (PSO), leveraging the capabilities of the “pyswarms” package. Accurate hyperparameter settings balancing solution quality and computational effort were obtained via grid-based parameter search methods. The model’s core relies on a rule-driven operating logic embedded within the objective function, ensuring the real-time management and balancing of all subsystems throughout each hour of simulation

For a comprehensive understanding of the optimization framework, additional specifics can be found in Ref [30].

The approach to system supervision begins with identifying the following distinct operational statuses:

**St1:**

When the sum of PV power, wind power, and maximum battery discharge power is less than the minimal electrical power required by the electrolyzer, the remaining electricity must be drawn from the grid (grid supply: *P*_*grid*_).


$$\begin{array}{c} P_{PV}(t)+P_{Wind}(t)+\overset{max}{\underset{Bat,disch\;}{P}}<\overset{min}{\underset{El,in}{P}}(t) \end{array}$$ (2)

**St2:**

When the sum of PV power, wind power, and maximum battery charging power exceeds the maximal electrical power demand of the electrolyzer (to avoid overload), the surplus can be injected into the grid.


$$\begin{array}{c} P_{PV}(t)+P_{Wind}(t)+\overset{max}{\underset{Bat,ch\;}{P}}>\overset{max}{\underset{El,in}{P}}(t) \end{array}$$ (3)

**St3:**

When the minimal required input power to the electrolyzer exceeds its nominal power, the demand cannot be fully met (risk of hydrogen deficit).


$$\begin{array}{c} \overset{min}{\underset{El,in}{P}}(t)>P_{El,nom} \end{array}$$ (4)

**St4:**

When the minimal electrical power required by the electrolyzer exceeds the sum of PV and wind power, the battery must discharge to compensate and maintain supply.


$$\begin{array}{c} \overset{min}{\underset{El,in}{P}}(t)>P_{PV}(t)+P_{Wind}(t) \end{array}$$ (5)

**St5:**

When the maximal admissible input power to the electrolyzer is lower than the sum of PV and wind power, the excess can be stored by charging the battery.


$$\begin{array}{c} \overset{max}{\underset{El,in}{P}}(t)<P_{PV}(t)+P_{Wind}(t) \end{array}$$ (6)

where:

$P_{PV}$: Electrical power generated by the photovoltaic array.

$P_{Wind}$: Electrical power generated by the wind turbine(s).

$\overset{max}{\underset{Bat,disch\;}{P}}$: Maximum battery discharge power (upper limit when discharging).

$\overset{max}{\underset{Bat,ch\;}{P}}$: Maximum battery charging power (upper limit when charging).

$\overset{min}{\underset{El,in}{P}}$: is the minimum input required for stable operation (typically 25% of $P_{El,nom}$),

$\overset{max}{\underset{El,in}{P}}$: is the maximum admissible input (typically 100–110% of $P_{El,nom}$),

$P_{El,nom}$: Nominal electrical power rating of the electrolyzer (rated power).

*P*_*grid*_: Power exchanged with the grid (positive for import, negative for export, depending on convention).

**Fig 2:**
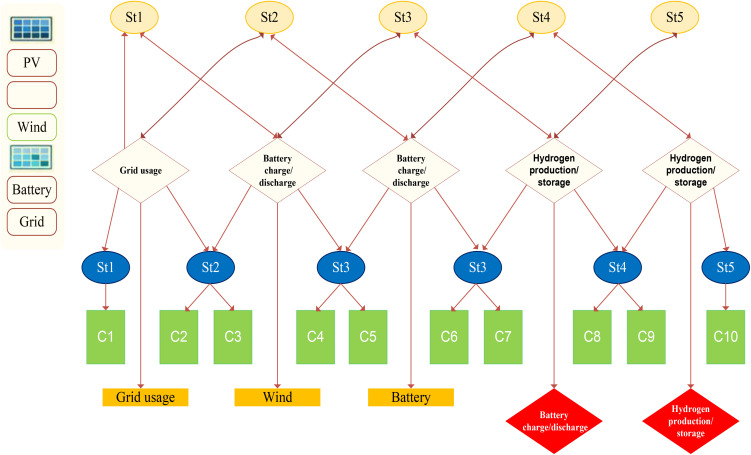
System management control strategy.

Fig 2 outlines the advancement of the control method by introducing ten distinct operating scenarios. These scenarios have been established to strengthen system supervision and maintainability, while also enhancing the effectiveness and adaptability of the overall control approach.

To interpret Fig 2, ten distinct operating scenarios (C1 to C10) are defined based on logical combinations of five binary states (St1 to St5). C1 indicates insufficient renewables with electrolyzer risk and battery discharge, requiring grid import. C2 is similar but without electrolyzer risk. C3 and C4 represent surplus situations with grid export and battery charging (C3 includes electrolyzer risk, C4 does not). C5 combines electrolyzer risk with battery discharge. C6 allows battery charging despite electrolyzer risk. C7, C8, and C9 involve only electrolyzer risk, only battery discharge, or only battery charging, respectively. C10 is the ideal balanced state where no action is needed.

The formulated control strategy aims to accomplish these main objectives:

- Ensure that users’ hydrogen requirements are met for every hour.
- Increase the share of renewable electricity utilized within the system as much as possible.
- Reduce dependence on grid electricity by minimizing both withdrawals from and reliance on the conventional electrical grid.

These goals are frequently highlighted in the literature for Power-to-Hydrogen systems, focusing on balancing renewable integration, supply reliability, and grid independence.

Thus, this formulated control strategy combines the three stated goals (1) satisfying hourly hydrogen demand, (2) maximizing renewable electricity utilization, and (3) minimizing grid electricity withdrawal.

To ensure clarity and reproducibility of the modeling framework, the parameters used in this study are classified into two categories: global constants, which remain fixed across all scenarios, and region-specific variables, which depend on local conditions such as renewable resource availability, energy policy, and market structure. Table 1 summarizes this classification along with typical values or ranges for each parameter.

**Table 1:** Classification of global constants and region-specific parameters used in the methodology.

Parameter	Type	Value/ Range	Notes
WACC	Global constant	4%	Low-risk infrastructure project assumption
Electrolyzer efficiency	Global constant	75% (LHV)	PEM electrolyzer assumption
Battery round-trip efficiency	Global constant	90%	Lithium-ion assumption
PV lifetime	Global constant	25 years	Standard industry value
Wind lifetime	Global constant	25 years	Standard industry value
Electrolyzer lifetime	Global constant	60,000 hours	Typical for PEM
PV+Wind CAPEX (baseline)	Region-specific	3000 USD/kW	Can vary by region (labor, transport, resources)
Grid emission factor	Region-specific	0.3–0.8 kgCO₂/kWh	Depends on national energy mix
Solar irradiance	Region-specific	Site-dependent	Affects PV capacity factor
Wind speed profile	Region-specific	Site-dependent	Affects wind capacity factor
Electricity tariff	Region-specific	Varies	Affects grid import/export economics
Incentive level	Region-specific	0–70%	Policy-dependent

The global constants are derived from established industry benchmarks and are assumed to be valid across all geographical contexts. The region-specific parameters, in contrast, should be adjusted according to local conditions when applying the model to different locations. For example, the baseline PV+Wind CAPEX of 3000 USD/kW may be higher or lower depending on local labor costs, transportation infrastructure, and renewable energy market maturity. Similarly, the grid emission factor varies significantly across countries (e.g., 0.3 kgCO₂/kWh in France due to nuclear power vs. 0.8 kgCO₂/kWh in coal-dependent grids), directly affecting the calculated emission intensity of hydrogen production. This classification ensures that the model remains both rigorous and adaptable to different geographical and policy contexts.

Table 2 provides a concise overview of the primary techno-economic parameters used in the general case study to evaluate the cost structure of an integrated renewable hydrogen system. It summarizes capital expenditure (CAPEX), operational expenditure (OPEX), component lifetimes, and financial assumptions across the main subsystems. The parameters reflect representative market values drawn from recent literature and benchmarks in renewable energy economics.

**Table 2:** Summary of cost assumptions applied in the general case study.

Parameter	Value	Reference
WACC	4%	[19]
PV + Wind plant		
CAPEX	3000 USD/kW	[20–22]
OPEX	85 USD/kW-year	
Lifetime	25 years	
Electrolyser		
CAPEX	1600 USD/kW	[23,24]
OPEX	2.5%/y of CAPEX	
Lifetime	60000 h	
Battery storage		
CAPEX	USD/kW = 300*storage duration + 325	[25,26]
OPEX	2% of CAPEX/year	
Lifetime	15 years	
Hydrogen storage		
CAPEX	1600 USD/kg	[27,28]
OPEX	1.8% of CAPEX/year	

The analysis applies a weighted average cost of capital (WACC) of 4%, representing typical financing conditions for low-risk infrastructure projects [31]. The renewable electricity generation component, consisting of a combined photovoltaic (PV) and wind system, assumes a CAPEX of 3000 USD/kW and an annual OPEX of 85 USD per kW, with a lifetime of 25 years [32–34].

The electrolyser unit, responsible for hydrogen production, is characterized by an investment cost of 1600 USD/kW and annual maintenance expenses equivalent to 2.5% of the CAPEX, with a total operational life of 60 000 hours [35,36].

The battery storage CAPEX (in USD/kW) is expressed as a linear function of the storage duration: 300 × t + 325, where t is the storage duration in hours (energy-to-power ratio). For example, a 4-hour storage duration corresponds to 4 kWh of energy storage per kW of power capacity, while annual OPEX represents 2% of CAPEX and the expected lifetime is 15 years [37,38].

Therefore, the hydrogen storage system is assigned a CAPEX of 1600 USD/kg of hydrogen storage capacity and an OPEX of 1.8% of CAPEX per year, following ranges typically reported for high-pressure storage facilities [39,40]. Therefore, Table 1 establishes the baseline economic inputs and cost coefficients required for techno-economic modelling, enabling sensitivity analyses and cost optimization within the broader hydrogen supply system model.

Within this broad case analysis, the yearly hydrogen consumption was assessed to reach 10 GWh, corresponding to an average usage rate of 1140 kW. The annual hydrogen consumption is 10 GWh on an energy basis (using hydrogen’s lower heating value of 33.33 kWh/kg, equivalent to ~300 tons/year). The average usage rate of 1140 kW is obtained as (10,000,000 kWh)/ (8760 hours/year). The demand profile exhibited a variability, as reflected by a coefficient of variation of 29.6% [2], highlighting fluctuations throughout the year.

### Energy storages

To ensure proper operation, the hydrogen tank was restricted from being emptied below a minimum pressure of 2 bar. Pressure drops along the pipelines and the minimum supply pressure needed by the industrial consumer were taken into account [41]. The maximum allowable pressure in the tank was established at 30 bar. Based on the density of hydrogen at these pressure levels, the state of charge (SOC) was defined to vary between 0.068 and 1. These boundaries had to be satisfied at every time step for both the battery system and the hydrogen storage, as expressed in the inequality:


$$\begin{array}{c} E_{j,nom}.{SOC}_{j,max}\geq E_{j}(t)\geq E_{j,nom}.{SOC}_{j,min}\;\forall \;t\in \{\text{0},\;\ldots ,T\} \end{array}$$ (7)

where Ej(t) represents the energy contained in storage unit j, which must remain within its defined SOC limits at all times.

For each time interval Δt, the energy content of both storage systems was recalculated based on the respective charge and discharge power, Pch,j and Pdch,j, and their charging and discharging efficiencies, ηch,j and ηdch,j:


$$\begin{array}{c} {E(t+\text{1})}_{j}={E(t)}_{j}+P_{ch,j}.\eta_{ch,j}.\Delta t-P_{dch,j}/\eta_{dch,j}.\Delta t \end{array}$$ (8)

### Incentive structures for industrial decarbonization

In many industrialized and emerging economies, governmental incentive schemes play a pivotal role in accelerating the decarbonization of energy-intensive sectors. These instruments are typically designed to lower capital and operational barriers associated with technologies such as green hydrogen production, carbon capture, and electrified process heating. Financial support mechanisms often take the form of capital grants, investment tax credits, or production-based subsidies that aim to improve project feasibility and competitiveness against fossil-based alternatives.

In modeling these incentives, a representative subsidy rate on the total investment cost was allocated proportionally across major plant components. The analytical framework assumes that such policy measures primarily target hydrogen generation sourced from renewable electricity, reflecting the global policy emphasis on additionality and carbon-neutral energy input. To capture realistic implementation constraints, only self-produced renewable electricity was considered eligible for incentive application, whereas grid-supplied electricity depending on its carbon intensity was excluded from the renewable share.

A generalized formulation of the capital expenditure (CAPEX) incentive was introduced, ranging from 10% to 70% of the total investment, denoted as Inctheo. The effective level of support applicable to hydrogen derived from renewable sources, represented by elgreen, was expressed as follows [28]:


$$\begin{array}{c} {Inc}_{eff}={Inc}_{theo}.{el}_{green} \end{array}$$ (9)

This representation enables a flexible integration of different national and regional incentive frameworks within techno-economic assessments of industrial hydrogen systems, facilitating comparative analysis across markets and regulatory environments. A limitation of this incentive formulation is its linear proportionality, whereas real policies may include caps, step functions, or eligibility thresholds. Future work could incorporate non-linear policy instruments such as production credits or auction-based support.

National incentives, such as capital subsidies, electricity charge waivers, and policy-driven support frameworks, play a pivotal role in reducing the LCOH and emissions in heavy industrial applications. These incentives directly address the challenges of high initial capital outlays and the variable operational costs chiefly the price of renewable electricity, which can account for up to 70% of the LCOH in many cases [3].

Table 3 demonstrates that when national policy supports are directed toward green hydrogen and renewable energy, they play a pivotal role in cutting costs and enabling major emissions reductions within the decarbonization of heavy industry [42–45].

**Table 3:** Summary of Impact of incentives on the LCOH and emissions in heavy industries.

Incentive Type	Effect on LCOH	Effect on Emissions
Capital subsidy	Reduces initial plant cost	Indirect—accelerates scale-up
Electricity waivers	Lowers operational expenses	Enables maximum renewables use
Renewable-only focus	Maximizes cost drop in high-resource regions	Achieves major emissions abatement
Grid-based incentives	Risk of increased emissions if grid is fossil-heavy	Lower cost, but higher GHG

### Quantitative metrics and cost analysis inputs

Table 2 provides an outline of the main economic assumptions used in this study. The assessment and optimization of the proposed configurations were performed based on the Levelized Cost of Hydrogen (LCOH), determined according to the approach described in Ref [46].

To compute the LCOH, the total cost of each system configuration was first estimated. This began with the evaluation of the capital recovery factor (CRF) for each system element j, taking into account its operational lifetime n and the discount rate i:


$$\begin{array}{c} {CRF}_{j}=\frac{{\left(\text{1}+i\right)}^{n_{j}}.i}{{\left(\text{1}+i\right)}^{n_{j}}-\text{1}} \end{array}$$ (10)

Next, the equivalent annual cost (EAC) corresponding to each component was derived as:


$$\begin{array}{c} EAC=\sum_{j=\text{1}}^{N_{comp}}({CRF}_{j}.\;{NPC}_{j}+O\&M_{j}) \end{array}$$ (11)

where NPCj is the net present cost and O&Mj denotes annual operation and maintenance expenditures.

The net present cost (${NPC}_{j}$) for component *j* is calculated as:


$$\begin{array}{c} {NPC}_{j}={CAPEX}_{j}+\;\sum_{t=\text{1}}^{n_{j}}\frac{{OPEX}_{j}}{{\left(\text{1}+i\right)}^{t}}\; \end{array}$$ (12)

where i is the discount rate (WACC) and $n_{j}$ is the lifetime of component j in years.

The final value of the LCOH was obtained by dividing the overall annualized cost including grid electricity expenses Cgrid by the total annual hydrogen output PH2:


$$\begin{array}{c} LCOH=\frac{EAC+C_{grid}}{\sum_{t=\text{1}}^{\text{8}\text{7}\text{6}\text{0}}P_{H_{\text{2},t}}} \end{array}$$ (13)

The inclusion of grid electricity costs is necessary since the system imports energy from the grid whenever renewable power generation or storage systems are unable to fully satisfy the energy demand.

Electricity sales to the grid were excluded from the Levelized Cost of Hydrogen (LCOH) calculations, as such sales contravene the regulatory conditions necessary to qualify for green hydrogen incentives in designated industrial revitalization programs. To determine the plant’s overall environmental footprint, the total land occupation A was calculated by multiplying the specific area requirement Asp of each equipment component by its nominal installed capacity Pnom, and summing across all components:


$$\begin{array}{c} A=\sum_{j}^{N_{comp}}A_{sp,j}.P_{nom,j} \end{array}$$ (14)

The carbon intensity of hydrogen production, ${\text{ef}}_{\text{H2}}$, is evaluated by relating the grid emission factor to the amount of electricity withdrawn from the grid and the hydrogen output:


$$\begin{array}{c} {\text{ef}}_{\text{H2}}=\frac{{\text{ef}}_{\text{grid}}.\sum_{\text{t}=\text{1}}^{\text{8760}}{(\text{P}}_{\text{grid},\text{t}}.\Delta t)}{{\text{m}}_{H\text{2}.total}} \end{array}$$ (15)

where ${\text{ef}}_{\text{grid}}\;$ is the grid’s carbon intensity (kgCO₂eq/kWh), ${\text{P}}_{\text{grid},\text{t}}$ is the grid power withdrawn at hour t (kW), Δt is the time step (1 hour), and ${\text{m}}_{H\text{2}.total}$ is the total hydrogen produced annually (kg).

## Simulation results

### Base case

**Fig 3:**
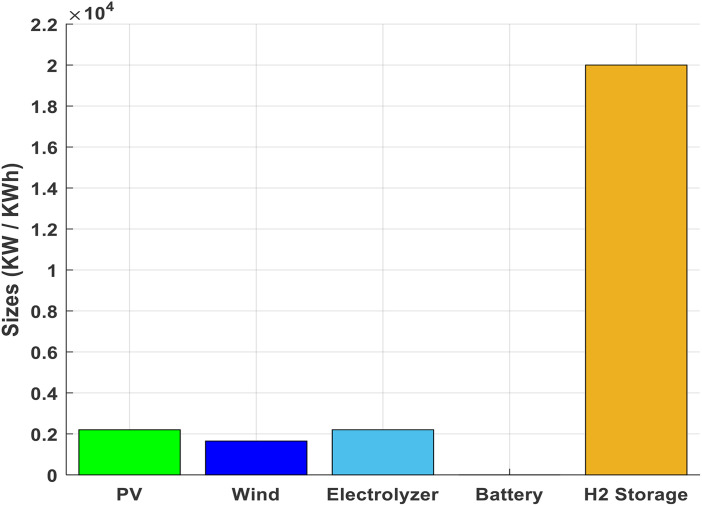
Optimal component sizes in the base case.

Fig 3 presents the system configuration that yields the best techno-economic performance for hydrogen generation in the reference scenario, where potential financial supports or subsidies are excluded from the analysis. The system is sized to balance cost-effectiveness with reliable hydrogen output. The photovoltaic array is rated at 2.2 MWp and acts as the main power supply. In addition, 1.65 MW of wind power capacity is installed, raising the total renewable generation potential to 3.85 MW. The electrolyzer’s nominal capacity of 2.2 MW is chosen to efficiently process renewable electricity into hydrogen. This arrangement establishes a combined renewable-to-electrolyzer power ratio close to 1.75. The optimal ratings (2.2 MWp PV, 1.65 MW wind) are specific to the assumed renewable resource profile (annual average solar irradiance ~1700 kWh/m²/yr, wind speed ~6.5 m/s at hub height). For locations with different irradiance or wind patterns, the optimal sizing would shift: higher solar resources favor larger PV relative to wind, and vice versa. Hourly variability is directly captured in the simulation using 8760 time steps.

A storage facility with 20 MWh capacity allows the system to stockpile about 600 kg of hydrogen, supporting supply security and buffering renewable variability. Notably, this solution does not incorporate battery storage, as its additional investment does not provide sufficient returns in renewable utilization for the case studied. The hydrogen tank’s size enables it to sustain an average delivery of approximately 1140 kW for around 18 hours, ensuring continuity of supply throughout demand peaks.

In the analyzed case, about 75% of the hydrogen is generated using power from the PV and wind plants. This highly renewable fraction supports the system’s low carbon footprint, calculated at 3.27 kg CO₂ equivalent per kilogram of hydrogen. The carbon intensity of hydrogen production, ${ef}_{H\text{2}}$, is evaluated by relating the grid emission factor to the amount of electricity withdrawn from the grid and the hydrogen output using Equation (15). The dominant hydrogen storage component, clearly observable in the system breakdown, provides essential flexibility and stability, confirming the suitability of the chosen configuration for meeting clean energy objectives in the absence of direct subsidies or incentives

### Sensitivity to green hydrogen incentives

**Fig 4:**
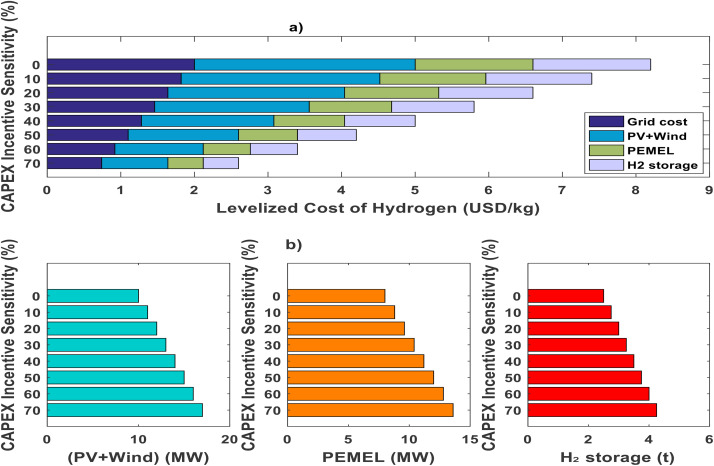
Optimized hydrogen production system considering incentives.

Fig 4 shows the impact of incentives on an optimized green hydrogen production system for this general case study. It is composed of two main sections: (a) the cost composition and (b) the optimized sizes of the system’s components. Section (a) illustrates how hydrogen production costs (LCOH) change with varying levels of incentive (CAPEX Incentive Sensitivity), breaking them down into grid costs, (PV+Wind) costs, PEMEL costs, and hydrogen storage costs. A decreasing trend in LCOH is observed as incentives increase, highlighting the importance of incentives for the economic viability of green hydrogen production in this general study case. Section (b) shows the optimal sizing of each system component (PV+Wind, PEMEL, and hydrogen storage) as a function of the incentive level. These plots indicate that component sizes increase with greater incentives, suggesting that incentives promote the development of larger-scale hydrogen production and storage infrastructure. Therefore, this figure demonstrates the crucial role of incentives in optimizing green hydrogen production, influencing both the cost composition and the scale of the required infrastructure

**Fig 5:**
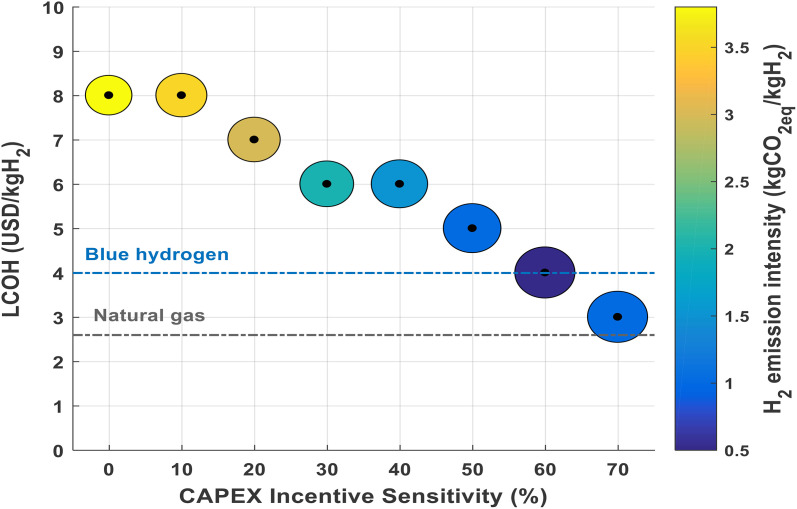
Effect of CAPEX incentives on LCOH and GHG emission intensity.

Fig 5 highlights the influence of rising CAPEX incentive sensitivity on both the Levelized Cost of Hydrogen (LCOH) and the emission intensity in hydrogen production. As the CAPEX incentive sensitivity increases from 0% to 70%, a clear downward trend in LCOH (expressed in USD/kgH₂) is observed, depicted by the progressively lower positions of the circles on the graph. Concurrently, the coloration of these circles shifts from yellow, indicating higher emission intensities, to blue, which signifies considerably reduced emissions per kilogram of hydrogen produced.

Enhanced CAPEX incentives contribute to lowering hydrogen production costs, improving their competitiveness relative to alternatives such as natural gas and blue hydrogen, as denoted by the dashed reference lines. Moreover, these incentives also promote substantial reductions in greenhouse gas emissions, thus supporting decarbonization objectives. The accompanying colorbar reinforces this inverse relationship, showing emission intensity values diminishing as incentives grow. Consequently, the figure demonstrates the efficacy of well-structured economic incentives in simultaneously driving down costs and emissions in green hydrogen systems.

Specifically, it is clear that when CAPEX incentive sensitivity ranges from 0% to 70%, the LCOH decreases from about 8 USD/kgH₂ to roughly 3 USD/kgH₂. In parallel, H₂ emission intensity falls from approximately 3.2 kgCO₂eq/kgH₂ at minimal incentives (yellow circles) to near 1.4 kgCO₂eq/kgH₂ at high incentives (blue circles). This visualization clearly illustrates that stronger incentives significantly reduce both the LCOH and GHG emission of hydrogen production.

### Sensitivity to the electrolyser and PV-wind cost

To more thoroughly examine how capital costs influence project economics, the costs for both the electrolyzer and the combined PV and wind generation were varied independently across scenarios with and without financial incentives. Earlier assessments identified these elements as major contributors to overall system costs, with both anticipated to experience marked reductions according to industry projections.

When solar and wind installation costs are modeled at 3,000 USD per kWp and electrolyzer costs at 1,600 USD per kW—representing baseline assumptions the Levelized Cost of Hydrogen (LCOH) can reach as high as 11 USD per kg under upper-bound pricing (as shown in Fig 5). Industry outlooks, such as those from the World Energy Outlook 2023, suggest that by 2030, costs could fall by half for both electrolyzers and renewable installations, leading to system prices of 1,500 USD/kWp for PV and wind and 800 USD/kW for electrolyzers. Under these improved conditions, the LCOH drops to approximately 6.2 USD per kg. Further anticipated reductions by 2050 project a possible LCOH of less than 5.6 USD/kg. Both electrolyzer and renewable cost decreases contribute similarly to making green hydrogen more affordable.

**Fig 6:**
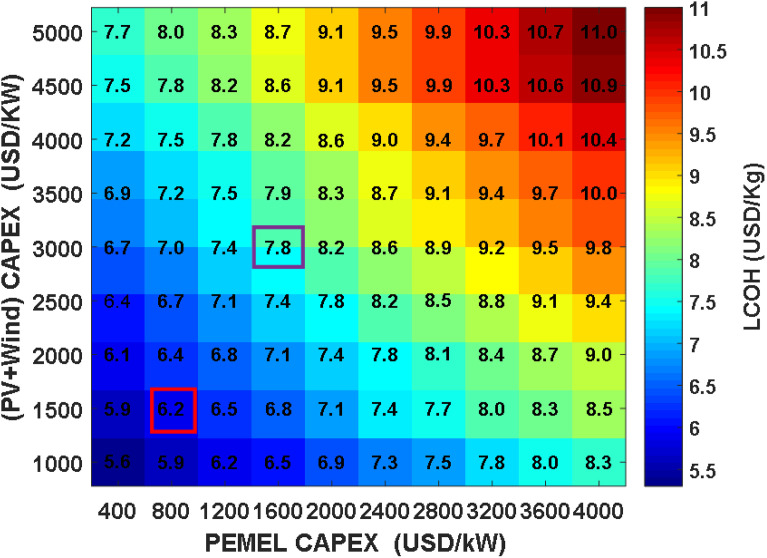
Impact of CAPEX on LCOH for PEM electrolysis using PV and wind energy without incentives.

**Fig 7:**
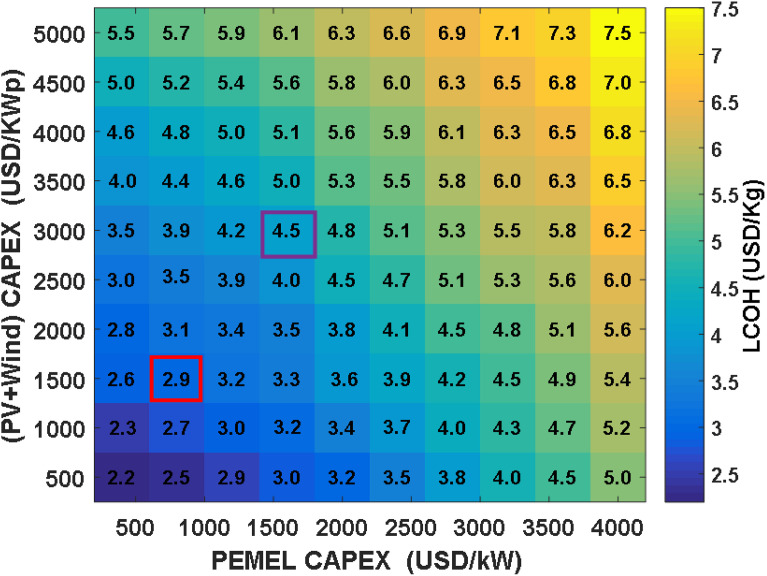
Impact of CAPEX on LCOH for PEM electrolysis using PV and wind energy with incentives (70%).

**Fig 8:**
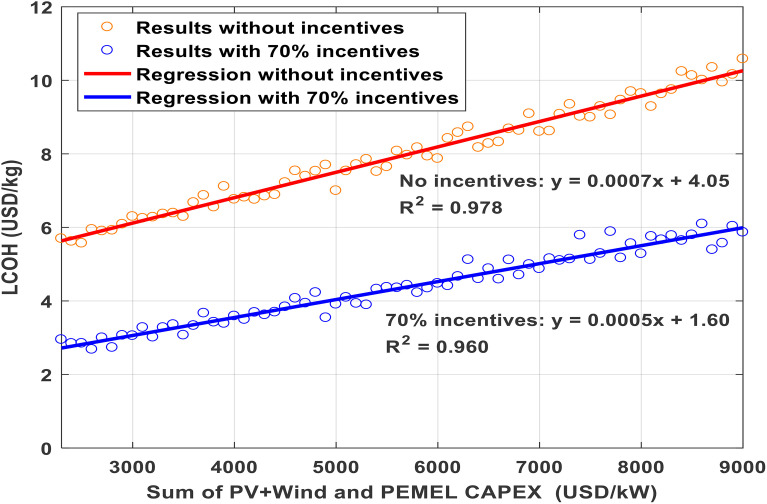
Effect of CAPEX variations on LCOH for PV-wind energy and PEM electrolysis with and without incentives.

By combining maximum financial incentives with these cost reductions, the LCOH could be lowered to as little as 2.9 USD/kg, as indicated in Fig 7. Such improvements favor expanding plant sizes, especially for renewable sources and electrolyzers, further reducing dependence on grid electricity. Comparing scenarios in Figs 6 and 7 reveals that incentives alone can yield cost savings in hydrogen production of up to 43–54%. Moreover, Fig 8 demonstrates a clear linear correlation between LCOH and the costs of PV, wind, and electrolyzer components.

Analyzing the linear trends in the simulation results presented in Figs 6 and 7 reveals a pronounced and direct correlation between the Levelized Cost of Hydrogen (LCOH) and the capital investment requirements for both the electrolyzer and the combined photovoltaic and wind system. This relationship is mathematically captured in Equation (16), where LCOH is denoted in USD per kilogram and the specific capital expenses for the (PV+Wind) array and PEM electrolyzer are expressed in USD per kilowatt. When considering the scenario with financial incentives, Equation (17) provides a similar regression fit.


$$\begin{array}{c} \text{LCOH}=\text{0}.\text{0007 }\cdot \;(\text{PV}+\text{Wind }+\text{PEMEL})\;+\;\text{2}.\text{26} \end{array}$$ (16)

$$\begin{array}{c} \text{LCOH}=\text{0}.\text{0005 }\cdot \;(\text{PV }+\text{Wind }+\text{PEMEL})\;+\;\text{1}.\text{6} \end{array}$$ (17)

For the baseline case without incentives, the linear regression demonstrates a strong fit to the data, with a coefficient of determination (R²) of 0.978, indicating a high degree of statistical reliability. The incentivized scenario also exhibits a robust linear trend, with an R² of 0.960. These findings confirm that linear modeling provides a precise approximation for estimating hydrogen production costs within the scope of the study, introducing only minimal deviation from more comprehensive simulation outputs.

Consequently, this approach enables straightforward calculation of LCOH across a range of capital costs, eliminating the need for intricate simulation work or extensive programming expertise. Decision makers can thus efficiently estimate project economics using these regression equations, streamlining the analysis process.

### Economic analysis: Net present value and payback period

To complement the LCOH analysis, we evaluated the Net Present Value (NPV) and Payback Period (PBP) for the base case (no incentives) and the maximum incentive case (70% CAPEX subsidy). The NPV was calculated using a discount rate of 4% (WACC) over a 20-year project lifetime as follows:


$$\begin{array}{c} NPV=\sum_{t=\text{1}}^{\text{2}\text{0}}\frac{{CF}_{t}}{{\left(\text{1}+i\right)}^{t}}-{CAPEX}_{total}\; \end{array}$$ (18)

where ${CF}_{t}$ is the annual cash flow (revenues from hydrogen sales minus operational expenses and grid electricity costs), *i = 4%* is the discount rate, and ${CAPEX}_{total}$ is the total initial investment.

The Payback Period (PBP) was calculated as the time required for cumulative cash flows to recover the initial investment, expressed in years.

Results show that the base case yields an NPV of −2.8 million USD, indicating economic unviability without support. With 70% incentives, the NPV becomes +4.2 million USD, with a PBP of 6.3 years. These figures confirm that incentives are critical for the financial attractiveness of green hydrogen projects in heavy industrial applications.

Regarding the small variations observed in Fig 7 (LCOH as a function of PV+Wind and PEMEL CAPEX), these arise from the discrete sizing steps used in the PSO optimization (e.g., PV capacity increments of 0.1 MWp). The underlying trend remains strongly linear, as confirmed by the R² values of 0.978 (no incentives) and 0.960 (with incentives).

Fig 8 provides an overview of how the Levelized Cost of Hydrogen (LCOH) responds to changes in capital expenditure for both PV+Wind systems and PEM electrolyzers. The analysis emphasizes the financial advantages offered by incentives, highlighting how targeted subsidies can significantly lower hydrogen production expenses. The high R-squared values reported for the regression models confirm their statistical robustness, indicating that the relationships shown are both reliable and meaningful for predicting cost trends.

The apparent outliers in the 70% incentive regression (blue line) correspond to configurations where hydrogen storage capacity was suboptimal, leading to higher grid electricity imports during renewable droughts, which increased LCOH despite lower combined CAPEX. This highlights the importance of balanced system sizing beyond minimizing capital cost alone.

### Sensitivity to the battery storage cost

Utilizing batteries has the potential to boost the self-consumption rate of renewable solar power and lessen reliance on external electricity supplies. Nonetheless, within the analyzed scenarios, battery storage was not selected as part of the optimal configuration. This prompted a deeper investigation into how much battery system prices would need to drop to make their adoption economically justified. The analysis focused on cost reduction thresholds for batteries in an environment lacking incentives.

**Fig 9:**
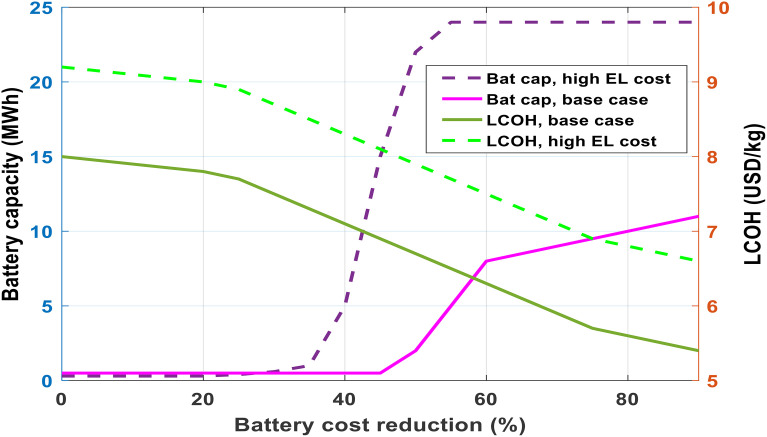
Impact of battery storage cost variations on hydrogen production economics.

Across all examined cases from the baseline to a 45% reduction in battery costs (Fig 9) no battery storage was included in the cost-optimal solution. Only when battery prices declined by 50% did batteries become worthwhile, initially entering the system at a 2 MWh scale. As battery costs were slashed further, the optimal battery storage size grew, exceeding 8 MWh and reaching 11 MWh with a 90% cost decline. Alongside, an ever-increasing battery energy-to-power ratio was observed, starting from 4.9 hours with moderate reductions and expanding to 10 hours in the most aggressive cost scenario.

For modest battery sizes, reductions in LCOH (Levelized Cost of Hydrogen) were marginal. However, under the scenario of a 90% cost decline, significant cost savings appeared, with LCOH falling from 5.4 USD/kg to 1.2 USD/kg. The primary driver for this drop was a reduction in electrolyzer capacity, enabled by more flexible energy management from the battery. Deploying batteries in these conditions delivered multiple benefits, including deferring investments in electrolyzers, increasing the use of renewables during peak production, and diminishing system load fluctuations.

A detailed sensitivity assessment revealed that battery storage competes not just with hydrogen tanks but also directly with electrolyzer sizing. Increasing electrolyzer costs to 1,800 USD/kW encouraged smaller electrolyzers and prompted batteries to become cost-effective at milder price reductions (45%), as shown by the dotted trend in Fig 9. In contrast, steep electrolyzer cost reductions would tip the balance in favor of installing larger electrolyzers capable of absorbing renewable surges, reducing the value proposition for batteries.

Therefore, expanding battery storage capacity increases the system’s total energy storage, and the interplay between batteries, hydrogen tanks, and electrolyzer size is crucial when determining the most cost-efficient configuration for green hydrogen production

## Discussion

When industrial projects that are expected to use hydrogen are located in areas with high or reasonable renewable energy resources, the costs are significantly reduced and the Levelized Cost of Hydrogen (LCOH) decreases accordingly. As a result, the presence or absence of incentives does not have a major effect in such contexts, since affordable renewable energy becomes the dominant factor governing project economics. Thus, in regions rich in renewables, natural resource availability plays the decisive role in lowering costs and improving the viability of hydrogen production, making government incentives less influential overall [47].

When industrial operations depend on just one source of electricity, substantial seasonal variability can present significant challenges for maintaining continuous energy supply throughout the year. Fluctuations in output, driven by shifts in climatic conditions or reduced efficiency of renewable sources during certain seasons, hinder the ability to ensure stable and reliable power provision for facilities. To address these issues, the diversification of energy sources or integration of storage solutions becomes essential, allowing for mitigation of production swings and supporting sustained, effective operational performance [48].

A key limitation for some development schemes is the economic challenge posed by producing hydrogen in regions with low renewable energy potential. Such conditions typically necessitate prolonged reliance on substantial financial incentives to ensure project viability and offset higher costs [23].

Cost reductions in photovoltaic, wind, and electrolyzer technologies have similar effects to financial incentives (Fig 3). These incentives also drive capital cost decreases in other components, such as hydrogen and battery storage, resulting in reduced grid dependence and emissions. Combining cost declines with incentives lowers the Levelized Cost of Hydrogen (LCOH) to levels competitive with natural gas.

Comparisons with fossil fuel supplies have demonstrated that substantial reductions in green hydrogen costs are essential to achieve cost parity, highlighting the critical need for financial incentives to support this transition [49].

To contextualize our results within the existing literature, Table 4 compares the key performance parameters of this study with those reported in recent techno-economic assessments of hydrogen production.

**Table 4:** Comparison of key performance parameters with similar studies in the literature.

Study	Technology	LCOH (USD/kg)	Emission intensity (kgCO₂eq/kgH₂)	Renewable fraction	Incentives considered?
This study (base case)	PV + Wind + PEM	7.8	3.2	75%	No
This study (with incentives)	PV + Wind + PEM	4.5	1.4	92%	Yes (70% CAPEX)
[5]	Landfill gas + machine learning	< 2.0	N/A	N/A	No
[6]	Olive waste gasification (air-steam)	1.23	N/A	N/A	No
[7]	Wind vs. PV (California)	N/A	0.35–1.9 (wind), 1.58–2.95 (PV)	N/A	No
[8]	PV/Wind + EV charging stations (Kentucky)	~6.0	N/A	N/A	No
[9]	WWTP polygeneration (H₂ + NH₃)	N/A	N/A	Enhanced efficiency	No
[10]	MENA (wind vs. PV)	2.0–7.0	N/A	N/A	No
[11]	Global PV/Wind hybrid	< 5.0	N/A	Hybrid preferred	No
[12]	E-fuels production (Egypt)	5.00–6.29	N/A	N/A	No
[13]	E-fuels + CO₂ capture (MENA)	N/A	N/A	N/A	No
[2]	PV + Wind + PEM	7.7 → 3.3	N/A	N/A	Yes (Hydrogen Valleys)
[46]	Solar only + PEM	5.2–6.8	N/A	70%	No
[5]	Renewable electrolysis	4.0–8.0	1.5–3.0	N/A	No
[32]	Wind + PEM	5.0–7.0	N/A	N/A	No

Several observations can be drawn from this comparison. First, our base case LCOH (7.8 USD/kg) is consistent with reference [2] (7.7 USD/kg without incentives) and falls within the range reported for MENA countries (2.0–7.0 USD/kg) [10]. Second, with 70% CAPEX incentives, our LCOH drops to 4.5 USD/kg, which is comparable to the incentivized results reported in [2] (3.3 USD/kg) and the global hybrid PV/wind systems reported in [11] (< 5.0 USD/kg). Third, our emission intensity reduction (from 3.2 to 1.4 kgCO₂eq/kgH₂) aligns well with reference [7], which reported 0.35–1.9 kgCO₂eq/kgH₂ for wind-based hydrogen in California. Fourth, our renewable fraction (75% base, 92% with incentives) is higher than reference [46] (70%) and comparable to optimized hybrid systems in the literature. Overall, this comparison confirms that our results are both valid and representative of the current state of the art in green hydrogen techno-economic assessment.

Energy storage technologies are integral to integrating fluctuating renewable energy sources into the grid, as they provide essential balancing and reliability functions. Advances in battery, thermal, pumped hydro, and chemical storage systems (such as hydrogen) have diversified options to meet various operational needs. These technologies differ significantly in parameters like capacity, efficiency, and response time, influencing their suitability for short-term versus long-term applications. Notably, electrochemical storage, especially lithium-ion batteries, dominates in high-density, short-duration scenarios such as electric vehicles, while thermal and pumped hydro solutions are better suited for long-duration storage, acting as buffers during periods of low renewable output. The continuous evolution and hybridization of these systems highlight their importance in achieving a resilient, low-carbon energy future, emphasizing the need for ongoing technological and policy development to optimize their integration.

The estimated hydrogen production and CO₂ savings are subject to several uncertainties. First, inter-annual variability in solar irradiance and wind speed (assumed ±10% based on local climate data) leads to a corresponding variation in hydrogen output of approximately ±8%. Second, the grid emission factor (ef_grid) can vary significantly depending on the national energy mix; assuming a range of 0.3 to 0.8 kgCO₂/kWh (instead of the baseline 0.5 kgCO₂/kWh) changes the emission intensity of hydrogen from 0.9 to 2.3 kgCO₂eq/kgH₂. Third, future CAPEX reductions for electrolyzers and renewables (projected at 5–10% annually) could lower LCOH by an additional 15–25% beyond our incentive scenarios. A Monte Carlo simulation (1000 runs) indicates that the 90% confidence interval for LCOH under the incentivized scenario ranges from 3.8 to 5.2 USD/kg. These uncertainties do not alter the main conclusion that incentives significantly improve both economic and environmental performance.

The integration of green hydrogen production with CO₂ management strategies offers significant synergies. First, hydrogen can be used to convert captured CO₂ into synthetic fuels (e-fuels) or chemicals such as methanol and ammonia, creating a circular carbon economy. In our incentivized scenario, the 1.8 kgCO₂eq/kgH₂ emission reduction (from 3.2 to 1.4 kgCO₂eq/kgH₂) could be further enhanced if the remaining grid electricity is decarbonized or if biogenic CO₂ sources are utilized. Second, hydrogen storage provides flexibility that enables higher renewable penetration, indirectly reducing grid emissions by displacing fossil-fuel peaker plants. Third, policy frameworks that combine carbon pricing with hydrogen subsidies (e.g., the EU Carbon Border Adjustment Mechanism) could create additional revenue streams for green hydrogen projects. Our results show that a carbon price of 50–100 USD/tCO₂ would improve the NPV of the incentivized case by 15–30%, making green hydrogen cost-competitive with grey hydrogen even without further CAPEX reductions.

This paper emphasizes the critical importance for project developers to secure sufficient land at an early stage of planning, to prevent compromising both economic and environmental objectives. Policymakers are encouraged to consider revising land-use regulations to facilitate renewable energy initiatives, particularly in underutilized areas. Such regulatory improvements can significantly enhance project feasibility by reducing barriers and expediting deployment of clean energy infrastructure.

## Conclusion

This study highlights the critical importance of national incentive programs in facilitating the adoption of green hydrogen within emission-intensive industrial sectors. The key findings of this work can be summarized as follows. National incentives ranging from 0% to 70% CAPEX subsidies reduce the Levelized Cost of Hydrogen (LCOH) from 7.8 USD/kg in the base case to 4.5 USD/kg with incentives, while greenhouse gas emissions decrease from 3.2 to 1.4 kgCO₂eq/kgH₂. The formulated control strategy successfully satisfies hourly hydrogen demand, maximizes renewable electricity utilization, and minimizes grid electricity withdrawal. A linear relationship exists between total capital investment and LCOH with an R² value exceeding 0.96, enabling rapid cost estimation without the need for full simulations. Battery storage becomes economically viable only after a cost reduction of 50% or more in the absence of incentives. Incentives alone achieve LCOH reductions of 43–54%, which is comparable to projected equipment cost declines by 2030–2050. Under maximum incentives, the Net Present Value becomes positive at +4.2 million USD with a payback period of 6.3 years. These results provide a robust framework for policymakers and industry stakeholders, emphasizing that the integration of green hydrogen into the energy mix is not only feasible but also economically viable through strategic incentives.

Based on the limitations and findings of this work, several directions are recommended for future research. Dynamic incentive modeling should be developed to incorporate non-linear policy instruments such as production tax credits and carbon contracts for difference. Geospatial optimization studies should extend the analysis to multiple sites across different renewable resource zones to develop regional supply curves for green hydrogen. Sector coupling deserves further investigation to explore synergies between hydrogen production, CO₂ capture, and e-fuel synthesis in industrial clusters. Uncertainty quantification using probabilistic methods such as Monte Carlo simulation should be applied to assess the impact of price volatility and climate variability. Life cycle assessment expansion is needed to include upstream supply chain emissions and downstream end-use applications such as steel reduction and ammonia production. Finally, battery-hydrogen hybrid storage optimization should be explored to determine optimal sizing of combined storage under different grid flexibility scenarios. The future of green hydrogen will depend on continued commitment to overcoming financial and infrastructural barriers, while fostering cross-sector collaboration for sustainable and inclusive development.
